# Dictogloss-based instruction and conjunction use in Palestinian tenth-grade EFL learners

**DOI:** 10.3389/fpsyg.2026.1935764

**Published:** 2026-09-18

**Authors:** Ahmad Jamil Abukhiran, Guliz Ozuturk

**Affiliations:** 1 Ministry of Education and Higher Education Palestine, Ramallah, Palestine; 2 Department of English language Teaching, Cyprus International University, Nicosia, Türkiye

**Keywords:** baseline adjustment, conjunctions, Dictogloss, EFL writing, matching familiar figures test, textual cohesion

## Abstract

**Introduction:**

The study aimed to examine differences in tenth-grade students' performance in the use of conjunctions between Dictogloss-based instruction and traditional instruction after controlling for pre-test performance, to examine the association of Matching familiar figures test (MFFT) response latency and error rate with post-test performance, and to explore their moderating role in the relationship between instructional method and post-test performance.

**Methods:**

Sixty Palestinian tenth-grade EFL students from two intact classes participated. One class received 45 Dictogloss sessions over 9 weeks, while the other received traditional instruction for the same duration. Performance was measured using a 20-item multiple-choice test covering coordinating, subordinating, and correlative conjunctions. ANCOVA-equivalent linear models adjusted for pre-test scores and included Pre × Method terms, HC3 robust standard errors, and Holm correction.

**Results:**

Supplementary analyses showed significant pre-to-post gains in the Dictogloss class but not in the traditional class; however, these within-group gains were interpreted descriptively. Primary analyses showed no significant adjusted Dictogloss–traditional differences after Holm correction, although significant Pre × Method interactions for coordinating, correlative, and total scores indicated variation according to pre-test performance. MFFT response latency and error rate showed no significant joint association with post-test performance and no significant moderation after Holm correction.

**Discussion:**

Overall, the findings support Dictogloss as a potentially useful structured approach for conjunction practice but do not establish its overall superiority to traditional instruction or a reliable moderating effect of MFFT performance.

## Introduction

Textual cohesion is a central component of discourse competence because it enables readers to perceive relationships among clauses, sentences, and ideas rather than treating them as isolated units (Council of Europe, 2001; Halliday and Hasan, 1976). Cohesion can be realized through lexical and grammatical resources; the present study focuses specifically on grammatical conjunctions. Conjunctions signal relations such as addition, contrast, cause, result, time, and condition, helping writers make relationships between propositions explicit. Their appropriate use contributes to textual connectedness, although cohesion should not be equated with coherence as a whole. Accordingly, conjunction use is treated here as a focused indicator of textual cohesion rather than a complete measure of writing quality.

Despite their functional importance, conjunctions remain difficult for many English as a Foreign Language (EFL) learners. Zahara et al. (2023) reported frequent cohesive-device errors involving conjunctions among secondary-level EFL students and linked these difficulties to factors such as insufficient functional knowledge, limited revision, overuse of familiar connectors, and impulsive responding. Such problems are particularly relevant where grammar teaching emphasizes rule recall more than the semantic relationships expressed in connected discourse. Selecting an appropriate conjunction therefore involves more than recalling a form: learners must identify the intended relationship between ideas and choose a connector that represents it accurately.

This decision-making requirement provides a rationale for considering conceptual tempo, or impulsivity–reflectivity, as an individual-difference variable. Conceptual tempo refers to the speed-accuracy pattern of responding when several alternatives are available: relatively rapid responding with more errors is associated with impulsive performance, whereas slower, more deliberate responding with fewer errors is associated with reflective performance (Kagan et al., 1964). In language-learning tasks, time devoted to analysis and response accuracy may matter when learners choose among competing grammatical alternatives (Michońska-Stadnik, 2013). Recent work also indicates that cognitive differences can contribute to variation in language-learning and writing-related performance (Estaji and Safari, 2023).

Dictogloss is theoretically relevant because it combines listening, note-taking, collaborative reconstruction, comparison, and correction. During reconstruction, learners must recover not only lexical content but also the logical relationships among ideas, discuss alternative formulations, and revise choices through peer interaction and comparison with the source text (Vasiljević, 2010). These processes can direct attention to the form-meaning relationship expressed by conjunctions and create opportunities to reconsider an initially selected connector. Research on modified Dictogloss and collaborative reconstruction has likewise shown that such tasks can promote attention to linguistic form and text organization during production (Lindstromberg et al., 2016; Lam and Aubrey, 2023).

Existing research supports Dictogloss as a language-learning and text-reconstruction activity, but the outcomes examined have often been broader than conjunction use itself. Studies have addressed writing accuracy, coherent text production, genre-related conventions, formulaic sequences, and other aspects of language learning, while research on individual differences suggests that learners may engage with Dictogloss tasks differently (Ishikawa and Révész, 2024). This provides a basis for examining learner-level cognitive-performance characteristics alongside conjunction outcomes.

Accordingly, based on the literature reviewed, the specific gap addressed by the present study is the limited evidence on Dictogloss-based instruction in which grammatical conjunctions are the directly measured outcome and learner variation in conceptual-tempo/MFFT performance is examined alongside the instructional comparison. This gap is particularly relevant at the secondary-school level and in the Palestinian EFL context. Rather than claiming that research on Dictogloss or cognitive style is absent, the study focuses on whether baseline-adjusted conjunction performance differs between Dictogloss and traditional instruction and whether the observed MFFT dimensions are associated with, or moderate, those outcomes.

The study was also motivated by classroom-based evidence. Drawing on his professional experience as an English teacher and direct work with tenth-grade students, the researcher observed persistent difficulty in the appropriate use of English conjunctions. A preliminary diagnostic focus-group session with 12 educational supervisors and English-language teachers supported the existence of the problem and identified possible contributing factors, including reliance on traditional approaches to grammar instruction. A subsequent expert session helped delimit conjunctions appropriate for the grade level, followed by textbook content analysis; procedural details are reported in the Method section. Together, the literature-based and classroom-based rationales provided the basis for examining Dictogloss as a structured approach to conjunction instruction while treating MFFT-related learner variation as exploratory.

### Aim and purpose

Accordingly, the present study examines differences in tenth-grade students' post-instruction performance in the use of conjunctions between Dictogloss-based instruction and traditional instruction after controlling for pre-test performance, and explores whether MFFT response latency and error rate moderate the relationship between instructional method and post-test performance. The binary impulsive/reflective grouping was retained only for supplementary analyses because its scoring rule could not be verified from the archived data as a joint function of response latency and errors.

### Objectives

To examine whether tenth-grade students' post-test performance in the use of conjunctions differs between Dictogloss-based instruction and traditional instruction after controlling for pre-test performance.

To examine whether MFFT response latency and error rate are jointly associated with students' post-test performance in the use of conjunctions after controlling for pre-test performance.

To explore whether MFFT response latency and error rate moderate the relationship between instructional method and students' post-test performance in the use of conjunctions after controlling for pre-test performance.

### Research questions

After controlling for pre-test performance, does tenth-grade students' post-test performance in the use of conjunctions differ between Dictogloss-based instruction and traditional instruction?

After controlling for pre-test performance, are MFFT response latency and error rate jointly associated with students' post-test performance in the use of conjunctions?

After controlling for pre-test performance, do MFFT response latency and error rate moderate the relationship between instructional method and students' post-test performance in the use of conjunctions?

### Hypotheses

**H01:** after controlling for pre-test performance, there is no statistically significant difference in students' post-test performance in the use of conjunctions between Dictogloss-based instruction and traditional instruction.

**H02:** after controlling for pre-test performance, there is no statistically significant joint association between MFFT response latency and error rate and students' post-test performance in the use of conjunctions.

**H03:** after controlling for pre-test performance, MFFT response latency and error rate do not significantly moderate the relationship between instructional method and students' post-test performance in the use of conjunctions.

## Theoretical framework

### Conjunction

As readers, we recognize that the quality of a text is not determined merely by the grammatical correctness of individual sentences, but by the writer's ability to establish clear relationships among ideas. How, then, are these relationships constructed within a text? Can cohesive devices, viewed as explicit linguistic markers, signal to the reader the type of connection between sentences (addition, causation, concession, result, etc.), thereby enhancing clarity and the smoothness of transitions? Or does the mere abundance of cohesive markers fail to guarantee textual unity unless they are employed appropriately and in accordance with their intended function? These questions place us at the heart of the relationship between cohesion and coherence.

Textual cohesion is defined as

“Cohesion in a text is created using formal links that connect sentences and clauses.” (Waller, 2015, p.31)

Halliday and Hasan (1976) identified five types of these links: reference, substitution, ellipsis, conjunction, and lexical cohesion. Coherence, on the other hand, is understood as the semantic/logical relationships that make a text comprehensible as an integrated whole to the reader (Witte and Faigley, 1981). However, Carrell (1982) criticizes the view of cohesion as a direct measure of coherence, arguing that while overt linguistic relationships may assist the reader, they alone do not explain how the overall meaning of a text is constructed. Similarly, Widdowson (1978) points out that the mere presence of numerous cohesive devices does not guarantee coherence; sentences may appear “linked” through connectors, yet the text may still fail to produce a meaningful whole. Therefore, it is insufficient to use cohesive devices abundantly; the writer must possess knowledge of their semantic functions and the rules governing their proper usage.

Related literature sometimes discusses conjunctions alongside connectors, connective adverbs, and the broader category of discourse markers; however, these labels are not fully synonymous. In general terms, conjunctions are linguistic items that link words, phrases, clauses, or sentences and signal semantic relationships between them (Gupta, 2018; Riggs, 2012). In the present study, the construct is restricted specifically to the grammatical conjunctions operationalized in the assessment—coordinating, subordinating, and correlative conjunctions. Broader discourse markers that fall outside these grammatical categories are not treated as study outcomes. Recent EFL research likewise treats conjunctions as cohesive devices that can be examined as a distinct instructional target (Alawerdy and Alalwi, 2022).

Although conjunctions are often introduced in grammar as coordinating, subordinating, and correlative conjunctions, their contribution to textual cohesion is fundamentally semantic rather than purely structural: they signal relations such as addition, contrast, cause–effect, temporal sequence, and condition. Effective conjunction use therefore requires sensitivity to grammatical form, semantic appropriateness, and context (Halliday and Hasan, 1976). In the present study, however, “ability to use conjunctions” is operationalized more narrowly as the ability to select the grammatically, semantically, and contextually appropriate conjunction in sentence and short-paragraph contexts on the 20-item multiple-choice test. The measure therefore assesses recognition/selection of appropriate conjunctions rather than extended free-writing production.

Halliday and Hasan (1976) view conjunction as a cohesive device through which units of text are linked via explicit connectors that reflect logical and semantic relations. Thus, conjunctions help readers interpret how one idea depends on or extends another, contributing to the cohesion of the text.

### Classification adopted in the study

To operationalize conjunction instruction and assessment in a way that aligns with the tenth-grade English curriculum in Palestine, the present study adopts a tripartite classification of conjunctions due to its commonality in instruction and its practicality for curriculum-based intervention and measurement. Conjunctions are classified as follows:

- Coordinating conjunctions: connect words, phrases, or clauses of equal grammatical rank.
- Subordinating conjunctions: introduce dependent clauses and connect them to independent clauses, indicating relations such as time, cause, condition, or contrast.
- Correlative conjunctions: occur in pairs to connect words, phrases, or clauses with parallel structure and equal weight.

### Target conjunctions in the Palestinian tenth-grade curriculum

Following the conjunction-selection procedure described in the Method section, the researcher analyzed the tenth-grade English textbook using the agreed conjunction list as the analytic framework. The target items identified in the relevant lessons were:

- Coordinating conjunctions: and, but, or, so, yet, for
- Subordinating conjunctions: because, although, if, when, while, as, since
- Correlative conjunctions: either…or, both…and, whether…or, as…as

The same curriculum-based set of conjunctions was taught to both the experimental and control groups during the same instructional period. The experimental group was taught through Dictogloss-based instruction, whereas the control group received traditional instruction.

### Conceptual tempo and MFFT performance (impulsivity/reflectivity framework)

The theoretical background for the MFFT component of the present study derives from the impulsivity/reflectivity framework described by Kagan et al. (1964), in which individuals differ in how they evaluate multiple alternatives before responding. Within this framework, an impulsive response pattern refers to responding quickly with relatively limited deliberation and a greater likelihood of error, whereas a reflective response pattern refers to slower, more deliberate evaluation of alternatives before a response is selected.

According to Kagan et al. (1964), this framework is operationalized through two primary performance dimensions: response speed and response accuracy. Impulsivity is theoretically associated with faster responding and more errors, whereas reflectivity is associated with relatively slower responding and fewer errors. In the present study.

The two styles also differ in several characteristics. Reflective individuals are described as tending to analyze and evaluate the likelihood of each option before selecting the most appropriate alternative, whereas impulsive individuals tend to provide rapid responses that may be prone to errors (Al-Khouli, 2002). Reflectivity is further associated with higher levels of caution and attentiveness, compared to impulsive individuals who may be more easily distracted and less focused (Abdul Jawad, 1990).

The study of cognitive styles is of considerable interest, as the personality-related aspects associated with them may interact with learning variables, memory, and information transfer (Messick, 1970). Additionally, the design of educational programs and assessment methods may be influenced, to some extent, by an individual's cognitive style (Al-Attoum, 2004). In the field of second language learning, Michońska-Stadnik (2013) demonstrated that the amount of time allocated to problem analysis and the potential number of errors during grammar acquisition are critical factors for understanding a linguistic task, making the impulsivity/reflectivity style worthy of attention. The term conceptual tempo is also used interchangeably with impulsivity/reflectivity to denote a cognitive style related to decision-making when handling linguistic tasks.

Recent language-learning research provides a more contemporary basis for considering learner-level cognitive variation. Banaruee et al. (2022), using a broader EFL learning-style framework, included the reflective–impulsive dimension among the learner characteristics examined in relation to reading performance, while Estaji and Safari (2023) examined impulsive/reflective learners in a writing-related instructional context. More directly, Chen et al. (2022) found that cognitive control became an important predictor of L2 grammar learning during the intermediate and later phases of learning, and Rivera et al. (2023) showed that executive-function differences were related to grammatical-rule learning in interaction with learning condition and rule difficulty. These studies do not establish an MFFT-based treatment effect in the present sample, but they provide a contemporary psychological and educational rationale for examining response latency and error rate as exploratory learner-level variables in language tasks requiring attention and linguistic decision-making.

Based on the literature, this dimension can be summarized as influencing the way information is processed during learning and problem-solving. Reflective individuals tend to engage in slower but more accurate processing, whereas impulsive individuals tend to process information more rapidly, potentially with less precision. Understanding these differences can help teachers design instructional approaches that better accommodate individual learner variations, thereby enhancing performance in language tasks that require accurate decision-making.

### Dictogloss strategy

Dictogloss is a collaborative, multi-stage strategy introduced by Wajnryb (1990), in which learners listen to a short text, take notes, and then reconstruct it collectively to produce a coherent text that closely approximates the original meaning. This strategy is classified as both a social and metacognitive strategy, as it contributes to input comprehension and its storage in working memory or long-term memory (Chiu, 2006; Lu, 2008). It is also associated with promoting cooperative learning, verifying understanding, and reducing learner anxiety (Habte-Gabr, 2006). Dictogloss is particularly relevant to the present study because, during text reconstruction, learners are required to retrieve the semantic and logical relationships between ideas (e.g., cause, contrast, addition, chronological sequence), providing a natural context for practicing the use of conjunctions as cohesive devices within the text (Vasiljević, 2010). This potential benefit is particularly relevant to conjunction use because reconstruction requires learners to infer semantic and logical relations among propositions and encode those relations through appropriate linking forms. Collaborative discussion and comparison with the original text create repeated opportunities to notice how particular conjunctions express addition, contrast, cause, condition, and temporal sequence. This mechanism is consistent with Lam and Aubrey (2023), who describe Dictogloss as directing learners' attention to lexical and grammatical features through discussion during joint text reconstruction.

Based on the literature reviewed for the present study, Dictogloss has been examined more often in relation to broader writing, genre, listening, or form-focused outcomes than in relation to grammatical conjunction selection as a distinct measured outcome, particularly alongside learner-level variation in conceptual tempo or MFFT performance. During a Dictogloss task, students listen, take notes, reconstruct text collaboratively, and apply semantic and grammatical knowledge, making the procedure theoretically relevant to the functional selection of conjunctions (Vasiljević, 2010).

Dictogloss follows a series of sequential, interrelated stages. Several studies that have implemented this strategy (e.g., Vasiljević, 2010; Marashi and Khaksar, 2013; Rahmi, 2016; Meteab, 2022; Jose, 2022) indicate that its application fundamentally adheres to the four stages outlined by Wajnryb (1990), which are:

1. **Preparation:** learners are introduced to the topic of the text, prior knowledge is activated, and instructions are clarified. Essential vocabulary may be provided if necessary, and students are then organized into groups.
2. **Dictation:** students listen to the text, usually twice: the first time for general understanding without writing, and the second time to note key words or ideas that will aid in the reconstruction stage.
3. **Reconstruction:** groups reconstruct a coherent text based on their notes, discussing wording, grammar, and textual links, with the aim of producing a text that closely approximates the original meaning.
4. **Analysis and correction:** groups compare their output with the original text or with other groups' versions, discuss their linguistic choices, and receive feedback and correction. Attention is drawn to recurrent errors, such as weak cohesion or incorrect use of conjunctions.

When implementing Dictogloss, the teacher's role primarily involves facilitation and organization, including preparing materials, managing the stages of the strategy, guiding discussions, and providing feedback and correction (Dewi, 2015; Xiu-lian, 2009; Yusri and Lestari, 2018). In this way, Dictogloss represents an appropriate instructional intervention for developing tenth-grade students' ability to use conjunctions within the curriculum content.

Within the theoretical impulsivity/reflectivity framework, the sequential structure of Dictogloss may create useful opportunities for different response tendencies. The separation of listening, note-taking, reconstruction, and analysis can provide faster, more error-prone responders with opportunities to pause, compare alternatives, and revise initial choices, while the reconstruction and comparison stages can also accommodate more deliberate response patterns. Peer discussion, feedback, and comparison with the original text further encourage reconsideration of conjunction choices. These mechanisms provide a rationale for the present exploratory analyses of continuous MFFT response latency and error rate.

### The relationship between the study variables

The literature shows that writing development in English language learners does not stop at sentence-level accuracy, but is closely linked to the learner's ability to construct a coherent text through logical and semantic relationships between sentences and ideas. Here, textual cohesion (cohesion) plays a key role as a network of linguistic links that helps the reader follow meaning throughout the text, among which conjunctions serve as a primary tool for signaling relationships such as addition, causation, contrast, and result. Accordingly, experimental studies have sought to test instructional strategies that support discourse-level text construction, foremost among them the Dictogloss strategy, which functions as a collaborative text-reconstruction task, offering opportunities for linguistic negotiation and attention to the relationship between form and meaning during production.

Kooshafar et al. (2012) provides direct evidence of the association between Dictogloss and improvements in writing, particularly in producing more coherent texts. The study examined the effectiveness of Dictogloss compared to explicit instruction, highlighting the role of cohesive devices in supporting textual consistency. This aligns with the view that enhancing writing involves strengthening the mechanisms that link sentences, including conjunctions within the cohesion system.

Similarly, Azmoon (2021) examined the effect of Dictogloss compared to Processing Instruction on writing accuracy among EFL learners, showing that Dictogloss was superior in enhancing accuracy. The study also noted that many experiments tend to measure accuracy as the primary indicator, while dimensions such as coherence and cohesion receive less attention in some contexts. This suggests that although evidence of Dictogloss's effectiveness exists, it is often assessed through general indicators (e.g., accuracy) rather than by measuring specific text-linking skills (such as conjunction use) as primary outcomes.

Furthermore, Alsamadani (2022) supports the idea that Dictogloss contributes to improving writing skills and learners' attitudes toward English learning. Using a quasi-experimental design, the study demonstrated improvements in favor of the experimental group, with a theoretical link to text-organization skills and the use of transitional elements and connectors in writing. Although the study implicitly aligns with the logic of “cohesive devices,” like many other studies, it does not treat conjunctions as a distinct, directly measured performance outcome.

Some experimental studies indicate that the value of Dictogloss is not limited to test outcomes but also lies in the linguistic attention it elicits during interaction. For instance, Mohamadi and Rahimpour (2018) examined how different types of collaborative tasks, including Dictogloss and text reconstruction, create opportunities for so-called meta-talk about discourse markers during task completion, showing that task design can direct learners' attention toward discourse-organizing devices. However, the focus of this study remains on “attention opportunities” (process) rather than on measuring improvements in the use of cohesive devices in the final text (product) as a specific dependent variable.

Findings from comparisons between dictation and Dictogloss support the idea that Dictogloss creates a rich learning environment through listening and reconstruction. For example, Yu et al. (2025) compared three listening-based activities, dictation, Dictogloss, and text-comprehension questions, with the aim of measuring the learning of multiword items, showing that task type affects learning outcomes. Although this study does not target cohesive devices as a direct outcome, it reinforces the notion that Dictogloss is not merely “dictation” but a reconstruction task that engages linguistic processing at a level beyond individual words or sentences.

In light of the growing recognition that the effects of instructional tasks are not uniform across learners, the literature indicates that individual differences may moderate the benefits of task-based interventions. In this context, Ishikawa and Révész (2024) provide evidence that learning through Dictogloss tasks can be influenced by individual factors such as language aptitude via the mechanism of written languaging, suggesting that task outcomes may vary according to learner characteristics. This provides a methodological rationale for extending research to cognitive-performance variables closely related to linguistic decision-making during writing, such as MFFT response latency and error rate. The traditional impulsivity/reflectivity framework provides the theoretical background for these dimensions, while the present study examines them empirically as continuous measures.

1. Within the literature reviewed for the present study, comparatively few experimental studies treat grammatical conjunctions as a central, directly measured outcome of a Dictogloss-based intervention. Much of the cited work instead emphasizes broader outcomes such as writing accuracy, general writing performance, coherent writing, genre conventions, or lexical/form-focused learning (Kooshafar et al., 2012; Azmoon, 2021; Alsamadani, 2022; Lam and Aubrey, 2023).
2. Evidence suggests that Dictogloss directs attention to discourse-organizing devices during task performance (e.g., discourse markers) through meta-talk; however, in many cases, this remains interpretive/procedural and does not translate into a direct measurement of performance improvement in the produced text (Mohamadi and Rahimpour, 2018).
3. A nascent line of research links Dictogloss with individual differences (e.g., aptitude) via written languaging, highlighting the need to extend this line toward learner-level cognitive-performance dimensions such as response latency and error rate on the MFFT, especially since the use of cohesive devices requires careful functional selection among multiple alternatives.

Accordingly, based on the literature reviewed, the gap addressed by the present study concerns the limited evidence on Dictogloss as an instructional approach for the three targeted categories of grammatical conjunctions—coordinating, subordinating, and correlative conjunctions—among tenth-grade Palestinian EFL learners, together with exploratory examination of whether learner variation in MFFT response latency and error rate is associated with, or moderates, baseline-adjusted post-test performance.

## Method

### Design

The study used a quasi-experimental pretest–posttest design with two intact classes. One class received Dictogloss-based instruction, while the other received traditional instruction. Teaching method constituted the instructional factor, whereas MFFT response latency and error rate were treated as continuous learner-level variables to examine their association with post-test conjunction performance and their potential moderating role in the relationship between instructional method and post-test performance, after controlling for pre-test performance.

### Context and participants

The study was conducted at Al-Arroub Secondary School for Boys in the northern Hebron governorate, Palestine, during the second semester of the academic year 2024/2025. The study population comprised all tenth-grade students in the school (*N* = 90). The study sample consisted of 60 tenth graders from two intact divisions. The school was chosen purposively, and two intact divisions were selected by a simple random draw. The two selected intact classes were then randomly assigned to the instructional conditions, with one class assigned to Dictogloss instruction and the other to traditional instruction. Thirty students were included in each class.

## Data sources

### Matching familiar figures test (MFFT)

The MFFT, originally developed by Kagan et al. (1964), was adapted to the study context (Appendix 1). It consists of 10 items, each presenting one standard figure and six similar alternatives. For each item, the student's first-response latency and the number of incorrect attempts made before identifying the correct match were recorded. Mean response latency and mean error rate were then calculated for each student. In the present study, these two measures represented the speed and accuracy dimensions of MFFT performance, respectively, and were treated as continuous learner-level variables. Lower response latency indicated faster responding, whereas fewer errors indicated greater response accuracy. The two MFFT dimensions were used to examine their joint association with post-test conjunction performance and their potential moderating role in the relationship between instructional method and post-test performance after controlling for pre-test performance.

### Conjunction use test

A 20-item multiple-choice test was developed to assess students' ability to use the conjunctions targeted in Units 7–12 (Appendix 2). The test covered three categories: coordinating conjunctions (8 items; 40%), subordinating conjunctions (7 items; 35%), and correlative conjunctions (5 items; 25%).

The selection of the target conjunctions followed a staged procedure. After the initial diagnostic focus-group session, the researcher conducted a second focus-group session specifically to determine which English conjunctions were appropriate for teaching to tenth-grade students. The researcher documented the notes from this second session and analyzed them qualitatively to identify points of agreement among the participants. The agreed-upon conjunction list was then used as the framework for a content analysis of the tenth-grade English textbook. This content analysis identified the relevant conjunctions and the textbook lessons in which they occurred. On this basis, a Teacher's Guide was developed for lessons incorporating the selected conjunctions, and the pre-/post-test was constructed using the same target conjunctions and relevant lesson content. Both the test and the Teacher's Guide then underwent a final evaluation by academic experts specializing in English language teaching methods, who confirmed the instruments' validity, clarity of instructions, and practical applicability in the classroom.

Statistical validity was examined through item–total and field–total correlations. Item analysis indicated a generally easy-to-moderate test. For reliability, the overall 20-item conjunction test showed acceptable internal consistency (Cronbach's α = 0.792) and split-half reliability of 0.787. The subscale alpha coefficients were more variable (α = 0.536–0.693).

Dictogloss Teacher's Guide. A teacher's guide was developed to standardize the Dictogloss intervention delivered to the experimental group (*n* = 30) and to ensure consistent implementation across lessons aligned with the tenth-grade English textbook (Units 7–12, second semester 2024/2025). the teacher's guide was designed to cover only the lessons that included the target conjunctions and to provide structured procedures for implementing Dictogloss in those lessons. Specifically, the guide included: (a) the validated conjunction set, coordinating conjunctions (and, but, or, so, yet, for), subordinating conjunctions (because, although, if, when, while, as, since), and correlative conjunctions (either…or, both…and, whether…or, as…as); (b) lesson objectives and the specific conjunctions to be practiced in each lesson; (c) selected texts/materials and step-by-step teacher directions for conducting the Dictogloss lesson according to Wajnryb's (1990) four stages (Preparation, Dictation, Reconstruction, and Analysis/Correction); and (d) detailed classroom procedures for grouping, time allocation, monitoring, and feedback.

### Operational definition of the conjunctions use variable

In this study, the ability to use conjunctions refers to tenth-grade students' capacity to select the appropriate conjunction to complete a sentence or short paragraph in English, expressing the intended relationship between two parts of a sentence or between sentences (e.g., addition, contrast/concession, causation, temporal sequence, condition, etc.). This is examined within Units 7–12 of the English textbook for the second semester.

This ability is operationally measured through:

- The total score obtained by the student on a multiple-choice test consisting of 20 items (duration: 40 min).
- Scoring: One point is awarded for each correct answer and zero for an incorrect answer, yielding a total score range from 0 to 20.
- Test content, which covers three types of conjunctions according to the specification table:

° Coordinating conjunctions (8 items = 40%)
° Subordinating conjunctions (7 items = 35%)
° Correlative conjunctions (5 items = 25%)

### Procedures

Dictogloss lessons followed the core stages described in the literature (Preparation, Dictation, Reconstruction, and Analysis/Correction), with students working collaboratively to reconstruct the text and discuss language choices, including appropriate conjunction use.

After obtaining the required administrative permissions, the instruments were piloted to establish validity and reliability. The main study procedures were: (a) administration of the MFFT before the intervention, with first-response latency and error counts recorded for each student; (b) administration of the Conjunction Use pre-test to all participants; (c) Dictogloss instruction for the experimental class and traditional instruction for the control class using the same textbook content (Units 7–12); and (d) administration of the Conjunction Use post-test.

Each group received 45 instructional sessions over 9 weeks (five sessions per week), with each session lasting 40 min. Separate sessions were allocated for the Conjunction Use pre-test and post-test. The researcher served as the instructor for both groups and was a secondary-school English teacher with 10 years of teaching experience. Both groups studied the same instructional content during the same period and under the same classroom conditions; the planned difference between the groups was the instructional method. The control group received the usual school instruction, which relied primarily on direct teacher explanation, teacher-centered presentation, rote learning and memorization, and explicit presentation of the rules governing conjunction use, without the Dictogloss procedures of listening and collaborative text reconstruction. For the experimental group, consistent implementation of the intervention was supported by the teacher's use of the researcher-prepared Teacher's Guide in every session and adherence to the four Dictogloss stages—Preparation, Dictation, Reconstruction, and Analysis/Correction—throughout the 45 instructional sessions.

### Data analysis

Conjunction scores were calculated from the 20 item-level responses (0 = incorrect, 1 = correct) for both the pre-test and post-test. The primary analysis of instructional method was conducted using an Analysis of Covariance (ANCOVA)-equivalent linear model for each conjunction outcome. Post-test performance was modeled as a function of the corresponding centered pre-test score, instructional method, and the Pre-test × Method interaction. Centering the pre-test score allowed the Method coefficient to represent the adjusted difference between Dictogloss-based and traditional instruction at the overall mean pre-test level, while the interaction term examined whether the relationship between pre-test and post-test performance varied according to instructional method. HC3 robust standard errors were used, and Holm correction was applied across the four conjunction outcomes to control for multiple testing.

For the MFFT analyses, standardized response latency and error rate were treated as continuous variables. Their joint association with post-test conjunction performance was examined after controlling for the corresponding pre-test score. To examine moderation, response latency and error rate were entered together with their interactions with instructional method, and joint robust tests were used to evaluate whether these MFFT dimensions moderated the relationship between instructional method and post-test performance. In addition, the binary impulsive/reflective classification was examined in a secondary analysis using a baseline-adjusted model that included instructional method, cognitive style, and the Method × Cognitive Style interaction. Independent-samples post-test comparisons and within-group paired pre–post comparisons were also reported as supplementary descriptive analyses. Primary statistical inference was based on the baseline-adjusted models, with statistical significance set at α =0.05.

Model assumptions were examined using appropriate diagnostic procedures. Homogeneity of variance for the supplementary independent-samples comparisons was assessed using Levene's test, while distributional assumptions were examined using Shapiro–Wilk tests. For the baseline-adjusted models, residual distributions and variance patterns were evaluated using Shapiro–Wilk and Breusch–Pagan tests. Because the diagnostic results indicated some departures from normality and non-constant variance across the outcomes, HC3 heteroskedasticity-robust standard errors were applied to the primary models to provide inference that was less dependent on conventional homoscedasticity assumptions.

Effect sizes and 95% confidence intervals were reported to complement statistical significance testing. For the primary baseline-adjusted analyses, 95% confidence intervals for the adjusted Dictogloss–traditional differences were calculated using HC3 robust standard errors and t-based inference. Standardized adjusted differences (d_adj) were obtained by dividing the adjusted Method coefficient by the pooled post-test standard deviation. For the supplementary comparisons, Cohen's d was calculated for independent-samples post-test differences and Cohen's d_z for paired pre–post changes. Partial eta squared (ηp^2^) was reported for the supplementary Method × Cognitive Style analyses. These effect-size estimates were used to describe the magnitude of the observed differences and associations alongside the primary baseline-adjusted findings.

### Ethics

Ethical considerations were observed throughout the study. The researcher sought formal permission from the Scientific Research Department of the Ministry of Education and Higher Education. The Department informed the researcher that a separate formal research approval was not required in this case because he was a Ministry employee, taught at the same school, and the participating students were already members of his regular classes. Permission to conduct the study was obtained from the school principal. Students were informed about the purpose and procedures of the study, and their voluntary assent to participate was obtained. Parental consent was not obtained because, according to the local procedural guidance communicated to the researcher for this classroom-based study, it was not required. Students' responses were treated confidentially, all data were anonymized and reported in aggregate form, and participants could withdraw at any time without penalty.

## Results

### Teaching method and post-test conjunction performance

Post-test scores were descriptively higher for the Dictogloss group than for the traditional-instruction group (Table 1). To examine differences between the two instructional methods while controlling for pre-test performance, baseline-adjusted linear models were estimated for each conjunction outcome. The models included the corresponding pre-test score, instructional method, and the Pre-test × Method interaction. HC3 robust standard errors and Holm correction were applied across the four outcomes.

**Table 1:** Baseline-adjusted analysis of teaching method (HC3 robust inference; Holm correction).

Outcome	Adjusted D–T difference at mean pre-test	Holm p (difference)	Holm p (Pre × Method)	Holm p (joint method terms)
Coordinating	0.086	0.439	0.014	0.002
Subordinating	0.047	0.457	0.127	0.097
Correlative	0.069	0.457	0.014	0.011
Total	0.067	0.439	0.033	0.011

D–T, Dictogloss minus Traditional. The adjusted difference is evaluated at the overall mean pre-test score. The Pre × Method term tests whether the association between baseline and post-test performance differs by instructional condition. The joint test evaluates Method and Pre × Method together.

Across the four outcomes difference between the Dictogloss and traditional-instruction groups at the mean pre-test level was statistically significant after Holm correction (adjusted *p* = 0.439–0.457). However, the Pre × Method term remained significant after Holm correction for coordinating, correlative, and total scores, indicating that a single uniform teaching-method effect does not adequately summarize these outcomes. The joint Method terms were significant for coordinating, correlative, and total scores, but because each method was represented by one intact class, these heterogeneous associations cannot be separated from class-level differences and should not be interpreted as a definitive causal Dictogloss effect.

The corresponding adjusted Dictogloss–traditional differences at the mean pre-test level, with 95% robust confidence intervals and standardized adjusted differences, were: coordinating = 0.086, 95% CI (−0.020, 0.192), d_adj = 0.341; subordinating = 0.047, 95% CI (−0.066, 0.160), d_adj = 0.184; correlative = 0.069, 95% CI (−0.045, 0.183), d_adj = 0.253; and total = 0.067, 95% CI (−0.021, 0.155), d_adj = 0.323. All four confidence intervals included zero, which is consistent with the absence of a statistically significant adjusted average teaching-method difference after Holm correction.

Supplementary effect-size estimates were calculated to describe the magnitude of the observed between-group and within-group differences. For the post-test between-group comparisons, Cohen's *d* values (Dictogloss minus traditional) were 0.265 for coordinating, 0.131 for subordinating, 0.341 for correlative, and 0.298 for the total score. Within the Dictogloss group, standardized pre–post changes (Cohen's d_z) were 0.783, 0.778, 0.542, and 1.122, respectively; the corresponding values for the traditional-instruction group were −0.053, 0.080, 0.000, and 0.006. For the supplementary Method × Cognitive Style analyses, partial eta squared (ηp^2^) values were 0.046 for coordinating, 0.098 for subordinating, 0.059 for correlative, and 0.111 for the total score.

### MFFT performance and baseline characteristics

Examination of MFFT performance showed substantial baseline differences between students classified as reflective and impulsive across the conjunction outcomes (Cohen's *d* ≈ 1.10–2.02) (Table 2). Response latency and error rate were strongly positively correlated (*r* = 0.902), indicating considerable overlap between the two MFFT dimensions. Accordingly, response latency and error rate were examined as continuous variables in the main MFFT analyses, while the binary reflective/impulsive classification was included as a secondary analysis.

**Table 2:** MFFT classification based on response latency and error rate.

Method	Reflective	Impulsive	Fast-accurate	Slow-inaccurate	Boundary/tie	Total
Dictogloss	2	1	11	11	5	30
Traditional	3	2	5	12	8	30
All	5	3	16	23	13	60

Diagnostic cut-points were the sample medians (latency = 9.9; mean errors = 0.4). Reflective = slower-than-median with fewer-than-median errors; Impulsive = faster-than-median with more-than-median errors. Exact median ties were flagged. This diagnostic rule is used to demonstrate why forcing all 60 students into two categories is not defensible from the archived measurements; it is not presented as a newly validated scoring standard.

The diagnostic classification based on both MFFT response latency and errors identified only eight students within the theoretically concordant reflective and impulsive categories (5 reflective and 3 impulsive). Given the limited number of cases in these categories, MFFT response latency and error rate were examined as continuous variables in the main analyses of their association with post-test performance and their moderating role in the relationship between instructional method and post-test performance. The binary reflective/impulsive classification was examined only as a supplementary analysis.

### Supplementary analysis of MFFT cognitive-style classification

After baseline adjustment, HC3 robust inference, and Holm correction, the main effect of the binary cognitive-style classification was not statistically significant for any of the four outcomes (Table 3). The Method × Cognitive Style interaction was not significant for coordinating or subordinating conjunctions, while the interaction for correlative conjunctions approached but did not meet the Holm-adjusted 0.05 significance criterion. For the total score, the Method × Cognitive Style interaction reached statistical significance (Holm-adjusted *p* =0.042); however, the homogeneity-of-regression-slopes assumption was not satisfied (*p* = 0.006). Therefore, the total-score interaction was not interpreted as evidence of a reliable moderating effect of cognitive style.

**Table 3:** Baseline-adjusted analysis by MFFT cognitive-style classification.

Outcome	Holm p: style	Holm p: Method × Style	Slope-homogeneity p	Interpretation
Coordinating	1.000	0.113	0.346	No significant style or interaction effect
Subordinating	1.000	0.113	0.763	No significant style or interaction effect
Correlative	1.000	0.053	0.364	Interaction not significant after Holm correction
Total	1.000	0.042	0.006	Slope-homogeneity assumption not met; interaction not interpreted

### Analysis of continuous MFFT response latency and error rate

Standardized MFFT response latency and error rate were entered simultaneously in the analyses for all 60 students (Table 4). After Holm correction across the four outcomes, the joint association of the MFFT dimensions with post-test performance was not statistically significant, and no significant Method × MFFT moderation effect was found. The smallest Holm-adjusted *p*-value for moderation was 0.085. Response latency and error rate were strongly correlated (r = 0.902; VIF ≈ 5.38 for each dimension); therefore, interpretation focused on the joint tests rather than on the separate coefficients. Overall, the findings provide no statistically significant evidence that MFFT response latency and error rate were jointly associated with post-test conjunction performance or that they moderated the relationship between instructional method and post-test performance.

**Table 4:** Joint tests of MFFT dimensions and method × MFFT moderation.

Outcome	Holm p: MFFT dimensions MFFT	Holm p: Method × MFFT moderation	Decision
Coordinating	1.000	0.462	No significant MFFT association or moderation
Subordinating	1.000	0.462	No significant MFFT association or moderation
Correlative	0.489	0.085	No significant MFFT association or moderation after Holm correction
Total	1.000	0.213	No significant MFFT association or moderation

### Decision on the study hypotheses

**H01:** after controlling for pre-test performance and applying Holm correction, no statistically significant adjusted difference in post-test conjunction performance was found between Dictogloss-based instruction and traditional instruction at the mean pre-test level. Therefore, H01 was not rejected. Significant Pre-test × Method interactions for coordinating, correlative, and total scores indicated that the relationship between instructional method and post-test performance varied across levels of pre-test performance; these interactions should be interpreted cautiously because each instructional condition was represented by one intact class.

**H02:** after controlling for pre-test performance and applying Holm correction, MFFT response latency and error rate showed no statistically significant joint association with students' post-test conjunction performance. Therefore, H02 was not rejected.

**H03:** after controlling for pre-test performance and applying Holm correction, no statistically significant joint moderating effect of MFFT response latency and error rate was found in the relationship between instructional method and students' post-test conjunction performance. Therefore, H03 was not rejected.

## Discussion

### R1. Teaching method after baseline adjustment

After controlling for pre-test performance and applying Holm correction, no statistically significant adjusted difference between Dictogloss-based instruction and traditional instruction was found at the overall mean pre-test level across the four conjunction outcomes. Although students in the Dictogloss group showed significant pre–post improvement in the supplementary within-group comparisons, these gains should be interpreted as evidence of improvement within that group rather than as evidence of a greater treatment effect relative to traditional instruction.

Significant Pre-test × Method interactions were found for coordinating conjunctions, correlative conjunctions, and the total score, indicating that the relationship between instructional method and post-test performance varied according to students' pre-test performance. Thus, the findings suggest baseline-dependent variation rather than a uniform advantage of Dictogloss across students. However, because each instructional condition was represented by only one intact class, instructional method was confounded with class membership. Consequently, the observed differences cannot be attributed exclusively to the instructional method.

The pedagogical rationale for Dictogloss remains relevant, as collaborative reconstruction can direct learners' attention to linguistic form while maintaining a communicative purpose (Vasiljević, 2010), and previous studies have reported benefits for broader writing outcomes (Kooshafar et al., 2012; Alsamadani, 2022). The present findings therefore support Dictogloss as a potentially useful instructional approach for practicing conjunction use, but they do not establish its general superiority over traditional instruction. Further research involving multiple classes within each instructional condition and appropriate control for pre-test performance is needed to determine whether its effectiveness varies according to learners' initial performance levels.

### R2. MFFT performance and cognitive-style indicators

MFFT response latency and error rate were examined as learner-level indicators related to response speed and accuracy. After controlling for pre-test performance and applying Holm correction, these two MFFT dimensions showed no statistically significant joint association with post-test conjunction performance. This indicates that variation in response latency and error rate was not reliably associated with students' adjusted post-test performance across the conjunction outcomes.

The supplementary analysis based on the reflective/impulsive classification similarly showed no statistically significant main effect of cognitive style across the conjunction outcomes after baseline adjustment and multiplicity correction. Moreover, substantial pre-test differences between the classified groups indicate that post-test differences should be interpreted cautiously, as they may partly reflect differences in prior performance rather than cognitive style alone. Overall, the findings do not provide sufficient evidence that reflective learners consistently outperform impulsive learners in conjunction performance.

### R3. Moderating role of MFFT performance

After controlling for pre-test performance and applying Holm correction, the joint Method × MFFT analysis showed no statistically significant moderating effect of response latency and error rate on the relationship between instructional method and post-test conjunction performance. The smallest Holm-adjusted moderation *p*-value was 0.085, indicating that the observed interaction patterns did not reach the adjusted significance criterion.

The supplementary analysis based on the reflective/impulsive classification also did not provide consistent evidence of moderation across the conjunction outcomes. Although the Method × Cognitive Style interaction for the total score reached statistical significance in the common-slope model, the homogeneity-of-regression-slopes assumption was not satisfied; therefore, this interaction was not interpreted as reliable evidence of moderation. Taken together, the findings do not support the conclusion that the relationship between instructional method and conjunction performance varies systematically according to MFFT performance or reflective/impulsive cognitive style.

### Implications

The findings support a cautious pedagogical use of Dictogloss as a structured activity for practicing conjunction use. Although the strategy provides learners with opportunities for listening, note-taking, collaborative reconstruction, comparison, and revision, the present study does not establish a uniform post-test advantage of Dictogloss over traditional instruction after controlling for pre-test performance. Dictogloss may therefore be used as one component of conjunction instruction rather than as an approach whose general superiority is established by the present findings.

The findings also do not support differentiating instruction solely on the basis of reflective/impulsive cognitive-style classification. Instructional decisions may be more appropriately informed by students' observed learning needs, initial performance, and formative classroom evidence. The Dictogloss procedures used in the study—including repeated listening, note-taking, collaborative reconstruction, comparison with the source or teacher-model text, corrective feedback, and opportunities for reflection—may nevertheless provide useful conditions for encouraging students to reconsider conjunction choices and attend more carefully to form–meaning relationships. Future research may examine whether additional supports, such as dedicated planning time, conjunction-choice checklists, and brief guided reflection activities, enhance these processes.

### Limitations and future research

Several limitations should be considered when interpreting the findings. First, the study involved only two intact classes, with one class representing each instructional condition; therefore, instructional method was confounded with class membership, limiting causal interpretation. Second, the reflective/impulsive groups differed substantially in pre-test performance, and the classification based jointly on MFFT response latency and errors resulted in very small numbers of students within the theoretically concordant reflective and impulsive categories. Consequently, findings based on the binary cognitive-style classification should be interpreted cautiously. Third, MFFT response latency and error rate were strongly correlated, which limits the interpretation of their separate contributions when entered simultaneously in the statistical models. Finally, the conjunction subscales contained relatively few items and showed variable internal consistency (α = 0.536–0.693); therefore, findings for the individual conjunction categories are less stable than those for the total score.

Additional limitations relate to the study design, sampling, implementation, measurement, and statistical testing. The students were not randomly assigned individually; rather, the two intact classes were randomly assigned to the instructional conditions. The sample was drawn from a single boys' secondary school, which limits the generalizability of the findings to female students, other schools, and different educational settings. Because the researcher served as the instructor for both groups, researcher expectancy and instructor-related implementation effects cannot be completely ruled out, although both groups received the same instructional content, instructional time, and classroom conditions. Implementation consistency was supported through the use of the researcher-prepared Teacher's Guide and adherence to the four stages of Dictogloss. However, no independent observer, formal fidelity checklist, or external implementation rating was used; therefore, independent verification of implementation fidelity was not available. The 20-item multiple-choice test primarily assessed students' recognition and selection of appropriate conjunctions and did not directly measure conjunction use in extended authentic writing. The pretest–posttest design may also have been susceptible to practice or familiarity effects. In addition, the intervention lasted 9 weeks and was followed by an immediate post-test without a delayed follow-up assessment; therefore, the durability of the observed gains cannot be determined. Finally, although Holm correction was applied across the four primary outcomes, multiple statistical tests were conducted, potentially increasing the likelihood of chance findings and therefore warranting cautious interpretation.

Future studies should use clearly defined and documented MFFT scoring and classification procedures, employ digital methods to record first-response latency where possible, and recruit sufficiently large samples to support adequately powered analyses of MFFT performance and cognitive-style variation. Multiple classes should also be included within each instructional condition to allow instructional-method effects to be distinguished from class-level influences. Statistical analyses should account directly for pre-test performance, examine relevant model assumptions, apply appropriate corrections for multiple testing, and report adjusted estimates together with confidence intervals. Longer intervention periods, delayed post-tests, and authentic writing-production measures of cohesion would further clarify whether Dictogloss contributes to sustained improvement in conjunction use and broader text construction.

## Conclusion

This study examined differences in tenth-grade Palestinian EFL students' performance in the use of conjunctions between Dictogloss-based instruction and traditional instruction after controlling for pre-test performance. It also examined the association of MFFT response latency and error rate with post-test performance and explored their moderating role in the relationship between instructional method and post-test performance.

Students in the Dictogloss group showed significant pre-to-post improvement in the supplementary within-group comparisons. However, after controlling for pre-test performance and applying HC3 robust inference and Holm correction across the four conjunction outcomes, no statistically significant adjusted difference between Dictogloss-based instruction and traditional instruction was found at the mean pre-test level. Significant Pre-test × Method interactions for coordinating conjunctions, correlative conjunctions, and the total score indicated that the relationship between instructional method and post-test performance varied according to students' initial performance. These patterns should be interpreted cautiously because each instructional condition was represented by only one intact class, making instructional method inseparable from class-level influences.

The MFFT analyses showed no statistically significant joint association between response latency and error rate and post-test conjunction performance after controlling for pre-test performance. Likewise, no significant moderating effect of MFFT response latency and error rate on the relationship between instructional method and post-test performance was found after Holm correction. Supplementary analyses based on the reflective/impulsive classification also did not provide consistent evidence of a cognitive-style effect or moderation.

Overall, the findings support Dictogloss as a potentially useful structured approach for practicing conjunction selection through listening, collaborative reconstruction, comparison, and revision. However, the study does not establish its general superiority over traditional instruction or provide sufficient evidence that its effectiveness varies according to MFFT performance or reflective/impulsive cognitive style. Future research should include multiple classes within each instructional condition, larger samples, clearly defined MFFT scoring procedures, independent implementation-fidelity measures, authentic writing-production tasks, and delayed post-tests to examine the durability and generalizability of the observed outcomes.

## Data Availability

The raw data supporting the conclusions of this article will be made available by the authors, without undue reservation.
