# Supplementary material for: Dictogloss-based instruction and conjunction use in Palestinian tenth-grade EFL learners
Source: Front Psychol. 2026 Sep 18;17:1935764. doi: 10.3389/fpsyg.2026.1935764 (PMC13630760; doi:10.3389/fpsyg.2026.1935764)
Supplement: Supplementary file 1 [file Supplementary_file_1.docx]

Conjunctions Use Test

Dear Student ___________________________

The researcher is conducting a study entitled “The Effect of the Interaction between the Dictogloss Strategy and the Cognitive Style in Developing Tenth Grade Students' Ability to Use English Conjunctions”, As a partial fulfillment of the PhD degree in English Language Teaching at Cyprus International University.

This test aims to measure your ability to use conjunctions included in Units 7-12 for the second-semester English textbook for grade 10.

Thus, the researcher humbly requests you to answer all the test items. Note: The outcome of this test will only be used for scientific research purposes and will not affect your academic performance or school grades.

Please read the following instructions carefully before you start:

- The time limit for this test is 40 minutes.
- The test consists of 20 items.
- Answer all questions.
- Choose only one answer for each question.
- Answer the questions carefully, one by one.

Thanks for helping!

Researcher: Ahmad Abukhiran.

Conjunctions Use Test.

Duration: 40 minutes.

Instructions: Select the most appropriate answer for every sentence.
____________________________________________________________

1. She enjoys oranges ………. apples.

a) for	b) but
c) or	d) and

2. It was raining, ………. I still wanted to go outside.

a) and	b) or
c) but	d) for

3. ………. you complete your homework, you are welcome to join us.

a) if	b) because

c) although	d) when

4. She studied diligently, ………. she passed the test.

a) yet	b) and
c) or	d) so

5. Ahmad stayed home ………. he was feeling ill.

a) although	b) if

c) because	d) when

6. The film was long, ………. it was enjoyable.

a) for	b) yet
c) so	d) and

7. “You would have been able to travel – though only by train: cars were ….. the rich”.

a) or	b) but
c) for	d) yet

8. I went to bed early, ………. I was tired.

a) but	b) yet
c) or	d) for

9. He was exhausted, ………. he kept working.

a) so	b) but
c) for	d) yet

10. “Say …………. you eat mostly things from the top, middle …… bottom of the pyramid”.

a) both ... and	b) whether ... or

c) either ... or	d) as ... as

11. “At one place, the train had to stop for three hours because ten …….. twelve thousand buffalo were walking slowly across the railway line!”

a) or	b) but
c) for	d) and

12. ………. I got to the station, I gave her a call.

a) if	b) while

c) although	d) when
13. “The carriages were free and started to slow down, ……. the engine now on its own went even faster”.

a) when	b) if

c) while	d) because

14. Mohammad is ………. tall ………. his friend Ali.

a) whether ... or	b) both … and

c) either ... or	d) as ... as

15. Students will leave the classroom ………. the bell rings.

a) although	b) when

c) while	d) because

16. You can eat ………. pasta ………. pizza for dinner.

a) both … and	b) whether ... or

c) either ... or	d) as ... as

17. She has an interest in ………. technology ………. science.

a) either ... or	b) both ... and

c) whether … or	d) as … as

18. “But let’s be positive. ……….. humans have never had to deal with so many problems before, we’ve never had so much technology to help us”.

a) because	b) if

c) although	d) when

19. **……….** you like your lecturer **……….** not, you must attend the lecture.

a) as … as	b) whether ... or

c) either ... or	d) both … and

20. ………. the sightseeing tour was interesting, it was too long.

a) when	b) if

c) while	d) because
