# Supplementary material for: Dictogloss-based instruction and conjunction use in Palestinian tenth-grade EFL learners
Source: Front Psychol. 2026 Sep 18;17:1935764. doi: 10.3389/fpsyg.2026.1935764 (PMC13630760; doi:10.3389/fpsyg.2026.1935764)
Supplement: Supplementary file 2 [file Supplementary_file_2.docx]

**Matching Familiar Figures Test**

**(MFFT)**

This test is made up of 10 sets of figures and is used to determine students' cognitive styles (reflective or impulsive) by matching figure.

There are seven figures in each item: one is the standard figure and the other six are similar, albeit with some small differences that change their appearance.

The test is administered one by one to each student in a suitable test room, which is quiet, well-lit and free from time pressure. First the student looks at the regular figure and is then asked to locate the correct figure that matches it from among the six figure cards.

For each item, the student's first-response latency was recorded. If the first response was incorrect, the student continued selecting from the alternatives in the same item until the correct match was identified. Each incorrect attempt was recorded.

Once the test is completed, the mean first-response latency and mean error rate were calculated for each student. In the present study, these measures were the speed and accuracy dimensions of MFFT performance and were analyzed as continuous variables for the learner level in the primary analyses. The reflective/impulsive classification was only used for additional analysis.

**Answer sheet**

Student name: …………………………. Section: ……

| **Correct answer number** | **Number of errors** | **Response time** | **Item** |
| --- | --- | --- | --- |
| **2** |  |  | Child |
| **3** |  |  | Map of Palestine |
| **5** |  |  | Al-Aqsa Mosque |
| **6** |  |  | Ibrahimi Mosque |
| **5** |  |  | Olive tree |
| **4** |  |  | Books |
| **6** |  |  | Cup of coffee |
| **1** |  |  | Bear |
| **5** |  |  | Rose |
| **5** |  |  | Helicopter |
